# Supplementary material for: Steering ecological-evolutionary dynamics to improve artificial selection of microbial communities
Source: Nat Commun. 2021 Nov 23;12:6799. doi: 10.1038/s41467-021-26647-4 (PMC8611069; doi:10.1038/s41467-021-26647-4)
Supplement: Supplementary file 1 — Supplementary Information File [file 41467_2021_26647_MOESM1_ESM.pdf]

Steering ecological-evolutionary dynamics to improve artificial selection of microbial communities

Equations and figures cited in Supplementary Figure captions can be found in the main text.

Supplementary Figures

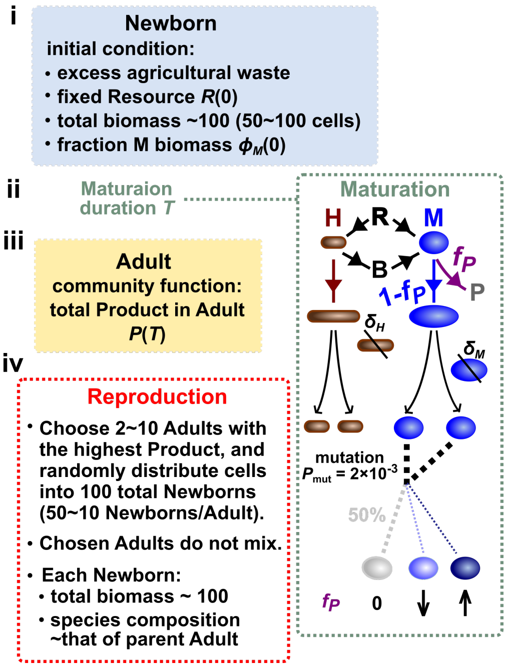

Supplementary Figure 1: A schematic of individual-based simulations of community selection over one cycle. During each selection cycle, $n_{\text{tot}} = 100$ communities mature from Newborns into Adults. **(i)** Each Newborn is supplied with excess agriculture waste and a fixed amount of Resource that supports the growth of 10^4 total biomass. Since agriculture waste is always in excess, it is not a state variable in Equations 1-5. The total biomass of each Newborn is approximately $BM_{\text{target}} = 100$. Since the biomass of individual M and H cells ranges between 1 and 2, the number of M and H cells in each Newborn is between 50 and 100. **(ii)** Maturation time is set to be short ($T = 17$, ~6 generations unless otherwise specified) to avoid Resource depletion (i.e. stationary phase in experiments) and cheater takeover. Each maturation cycle is divided into time steps of length $\Delta\tau = 0.05$. During each time step, the biomass of each M and H cell grows according to Equations 4 and 5 while the concentration of Resource, Byproduct and Product change according to Equations 1-3. At the end of each $\Delta\tau$, each M and H cell dies with a probability of $\delta_M \Delta\tau$ and $\delta_H \Delta\tau$, respectively. If a cell's biomass exceeds the threshold of 2, the cell divides into two identical daughter cells. Each daughter cell then mutates with a probability of $P_{\text{mut}} = 2 \times 10^{-3}$. In most simulations, only f_P of M mutates while other phenotypes are held at their bounds whose values are shown in the "Bounds" column of Table 1. In Figure 7, M's f_P, g_{Mmax}, K_{MR} and K_{MB} mutate independently, and H's g_{Hmax} and K_{HR} mutate independently. If a mutation occurs, it could be a null mutation with a probability of $\frac{1}{2}$. A null mutation reduces f_P, g_{Mmax} and g_{Hmax} to zero, while increases K_{MR}, K_{MB} and K_{HR} to infinity (equivalent to reducing affinities to zero). If a mutation is not null, it modifies each phenotype by ~5%-6% on average. **(iii)** At the end of a maturation cycle, community function is quantified from the amount of Product P in the Adult, $P(T)$. In some simulations, measurement noise is added to the true $P(T)$ to yield the measured community function. **(iv)** At the end of the selection cycle, top 2 or 10 Adult communities with the highest measured function are chosen to be reproduced into Newborns with different methods ("sorting" or "pipetting"). In all reproduction methods, the dilution factor of a chosen Adult is adjusted so that the biomass of Newborn communities is on average $BM_{\text{target}} = 100$. Justification of all parameter choices is discussed in detail elsewhere[1].

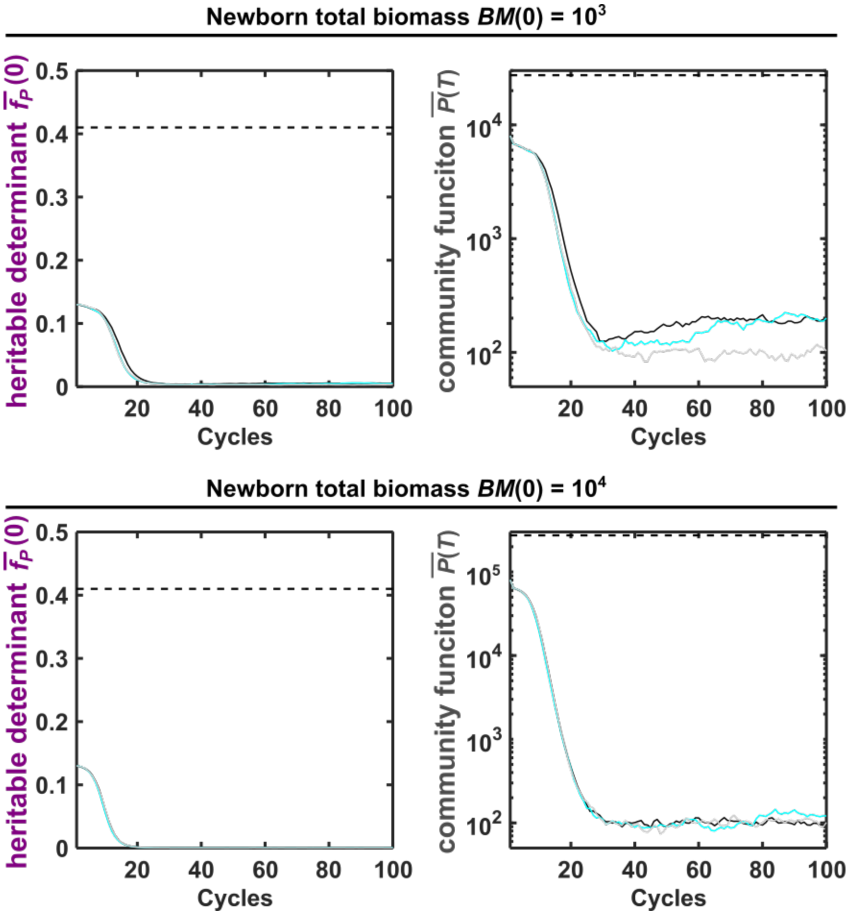

Supplementary Figure 2: Large population size interferes with community selection. Evolutionary dynamics of artificial community selection when the Newborn volume and thus total biomass are scaled up by 10 times (top panels) and 100 times (bottom panels) compared to simulations shown in Figure 6c. As a result, the average total biomass in a Newborn community are 10^3 and 10^4, respectively. Since cheaters take over more deterministically in large populations, f_P and community function decline to very low levels within 40 cycles, although they are maintained at positive values due to inter-community selection. Black, cyan and gray curves represent three independent replicates. Dashed lines mark f_P^* optimal for community function or the maximal community function.

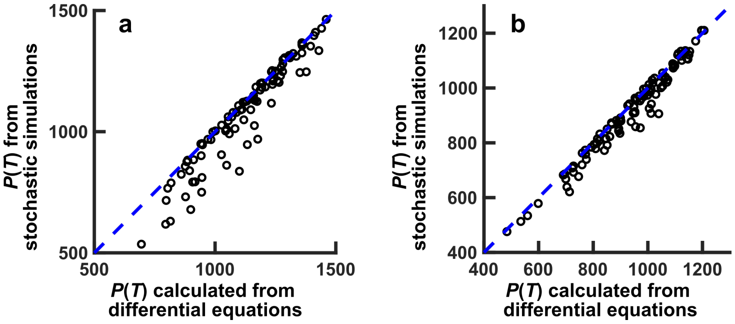

Supplementary Figure 3: Community functions $P(T)$ obtained from stochastic simulations can be adequately predicted by numerically integrating deterministic differential Equations 1-7. (**a**) We calculated community function $P(T)$ for 100 communities from the 2000th cycle of the simulation plotted in black curves in Figure 6c, using Equations 1-7 with $\phi_M(0)$ as the initial condition and $\overline{f}_P(0)$ as the f_P parameter. We then compared calculated $P(T)$ with $P(T)$ from stochastic simulations, and observed a reasonable concordance. Note that community functions from simulations (where cheaters M cells with small cost f_P increase in frequency) are generally lower than those from calculations. (**b**) We calculated $P(T)$ of 100 communities from the 2000th cycle of the simulation shown in black curves in Figure 7(a-b) with differential equations. For each community, we used its Newborn total biomass and Newborn species composition as initial conditions and the following within-Newborn averages as parameters: average M's cost $\overline{f}_P(0)$, average maximal growth rates $\overline{g}_{Hmax}$ and $\overline{g}_{Mmax}$, average affinity of M to Byproduct $\overline{K}_{MB}$, average affinity of M to Resource $\overline{K}_{MR}$, and average affinity of H to the Resource $\overline{K}_{HR}$. Calculated communities functions agree well with those obtained from stochastic simulations.

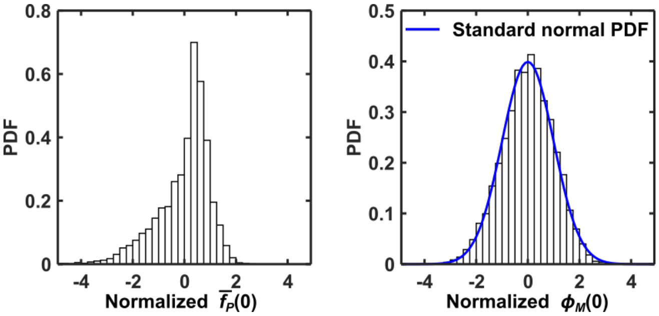

Supplementary Figure 4: Probability density functions (PDF) of normalized $\overline{f}_P(0)$ and $\phi_M(0)$ of offspring communities plotted in Figure 2e. Determinants are normalized within lineage before pooled together to yield the PDFs. Specifically, if the determinant of the jth offspring community descending from the ith parent community is denoted as x_{ij}, the normalized determinant is $\hat{x}_{ij} = (x_{ij} - \overline{x}_i)/\sigma_i$ where $\overline{x}_i$ and σ_i are respectively the mean and standard deviation of the determinants obtained from all offspring communities descending from the ith parent community. If the within-lineage distribution of x_{ij} is normal, then the pooled distribution of $\hat{x}_{ij}$ satisfies a standard normal distribution, as is the case for normalized $\phi_M(0)$. This is not true for normalized $\overline{f}_P(0)$, whose distribution is skewed. Both PDFs are obtained from ~6000 offspring communities (100 parent communities × ~60 offspring communities per parent community).

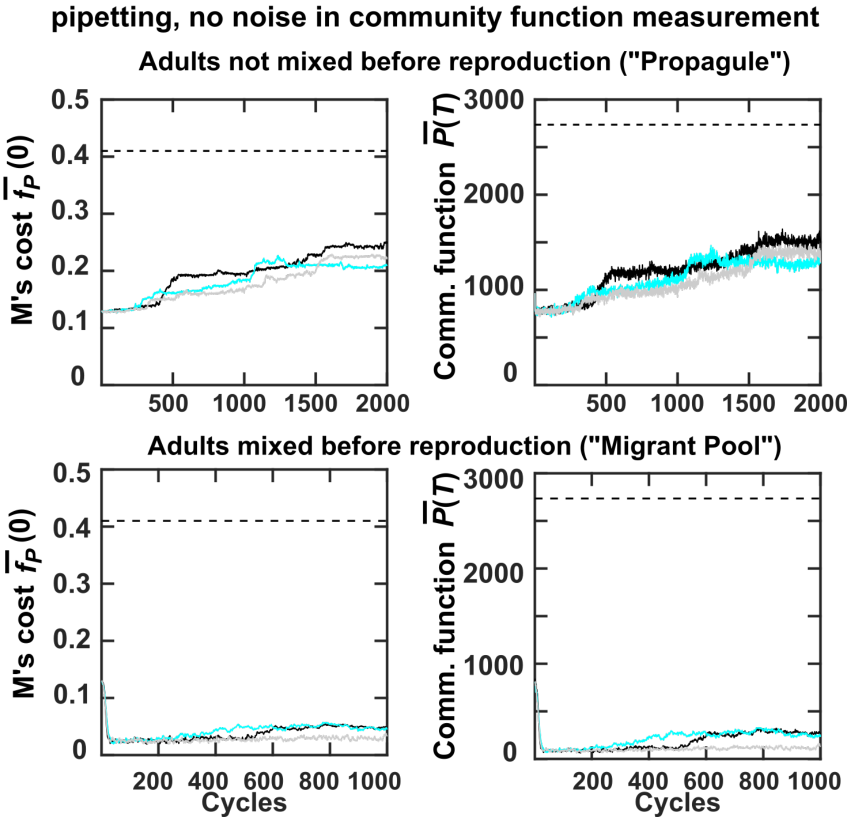

Supplementary Figure 5: Mixing Adults before reproduction interferes with selection. Evolutionary dynamics of artificial community selection when chosen Adult communities are not mixed (top) or mixed (bottom) before reproduction. Top 10 communities were chosen for reproduction via pipetting so that Newborn total biomass and species composition stochastically fluctuated. Measurement noise was not considered. Top (replicate runs of Figure 3 G and H from our previous work[1]): Adults were not mixed before reproduction, and each gave birth to 10 Newborns. Bottom: Adults were mixed, and the mixture was randomly split into 100 Newborns. Figure legends are the same as Supplementary Figure 2.

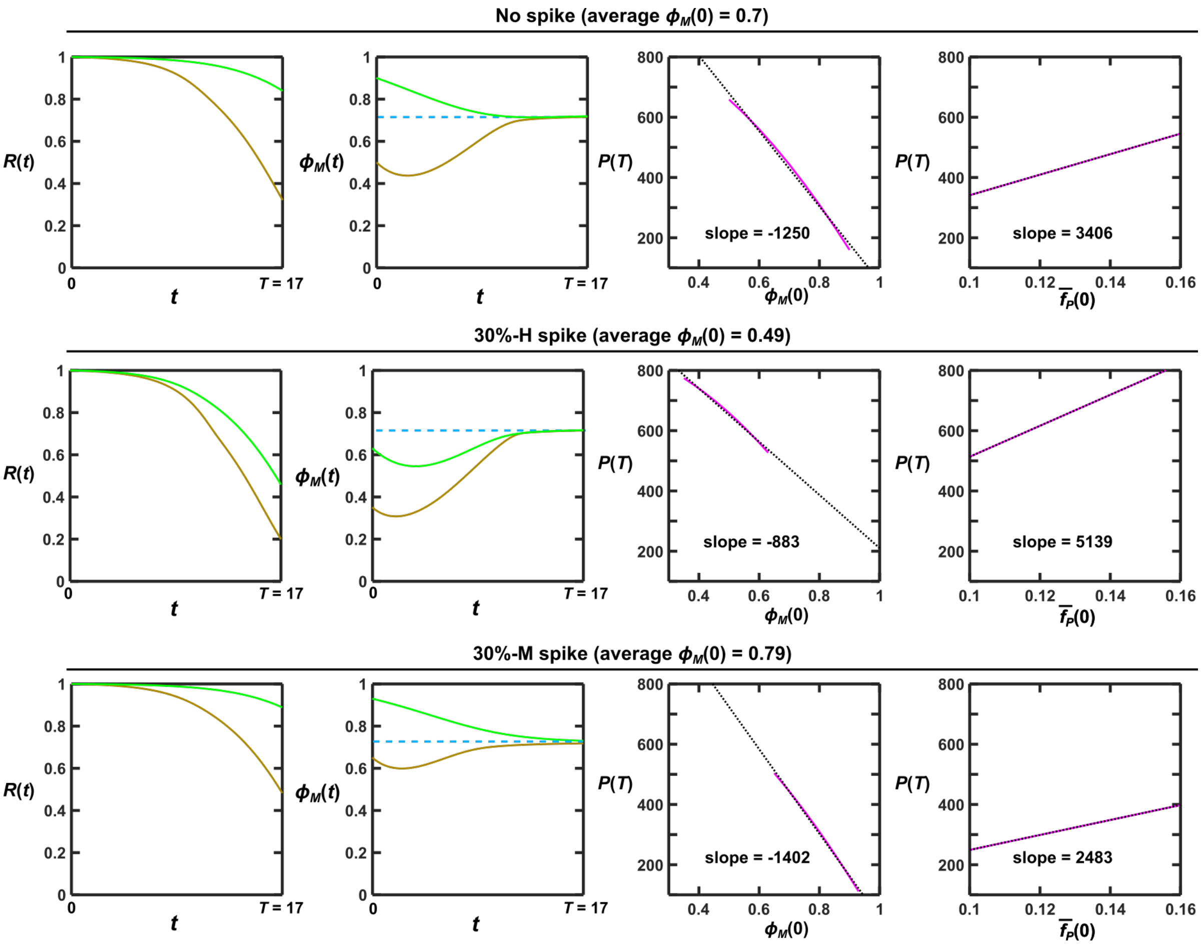

Supplementary Figure 6: Species spiking alters how strongly community function is impacted by heritable and nonheritable determinants, despite the erase of spiking effect during community maturation. We compare three spiking strategies: no spiking (top row), 30%-H spike (middle row), and 30%-M spike (bottom row). **First and Second columns**: dynamics of Resource and of $\phi_M(t)$ over one cycle when $f_P = 0.13$ and when $\phi_M(0)$ is high (green curve) and low (mustard curve). The second column demonstrates that the effect of spiking is erased within one cycle, since all curves reach the same steady state (blue dashed line) dictated by the blue attractor in Figure 2b. **Third column**: dependence of community function $P(T)$ on the nonheritable determinant. These three columns are computed by integrating Equations 1-7 assuming $f_P = 0.13$. **Fourth column**: dependence of community function $P(T)$ on the heritable determinant. The three panels are computed by integrating Equations 1-7 with $\phi_M(0) = 0.7$ (no spiking), $\phi_M(0) = 0.7\times(1-0.3) = 0.49$ (30%-H spiking), and $\phi_M(0) = 0.7\times(1-0.3)+0.3 = 0.79$ (30%-M spiking), respectively. In the third and fourth columns, the slope of each magenta curve is obtained from least square linear regression. A larger absolute value of slope reflects a stronger dependence of $P(T)$ on the determinant. Notice that under 30%-H spiking, the dependence of community function $P(T)$ on the nonheritable determinant $\phi_M(0)$ is the weakest, while the dependence on the heritable determinant $\overline{f}_P(0)$ is the strongest. This means that for given variations in $\phi_M(0)$ and $\overline{f}_P(0)$, the heritability of community function is the highest under 30%-H spiking.

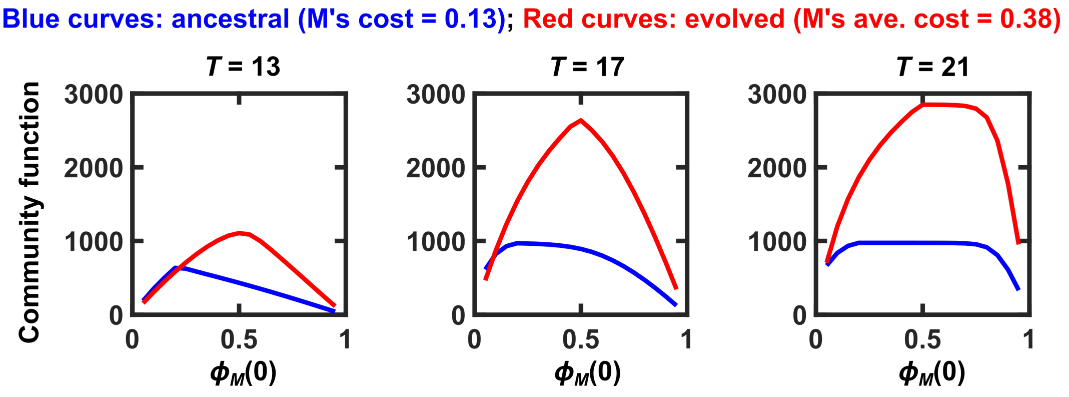

Supplementary Figure 7: The function of an evolved community ($\overline{f}_P(0) \approx 0.38$ achieved by Cycle 1000 in Figure 6 b and d) is higher than that of an ancestral community ($f_P(0) = 0.13$) under various maturation times and Newborn species compositions. Here, community functions are calculated from Equations 1-7 assuming $BM(0) = 10^2$ under various $\phi_M(0)$.

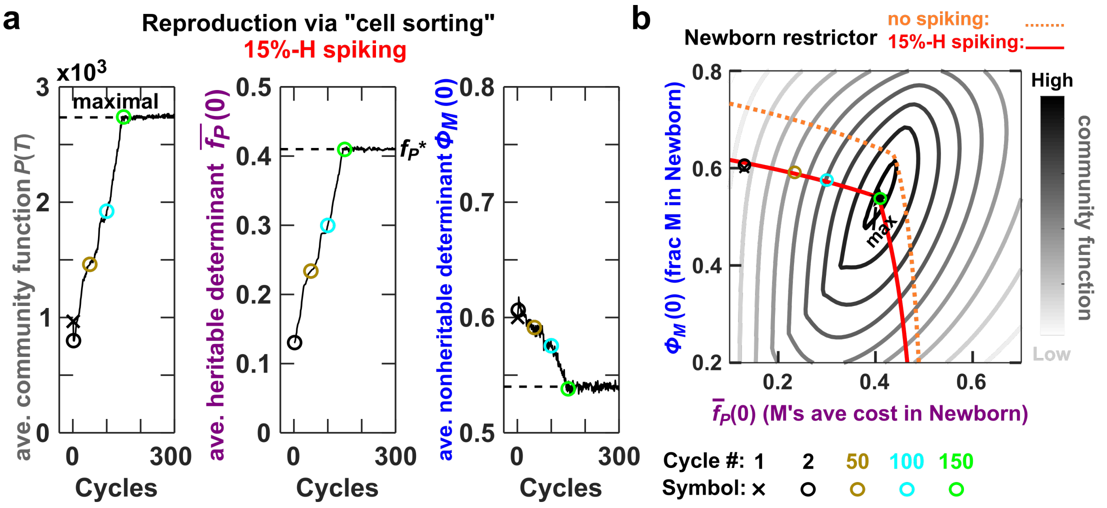

Supplementary Figure 8: Community function can reach the global maximum under a proper spiking strategy. **(a)** The evolutionary dynamics of the H-M community where chosen Adults are reproduced via cell sorting under 15%-H spiking: each Newborn receives 85% of its biomass from its parent Adult and the rest 15% is made up of H cells (which do not evolve). **(b)** Compared to the Newborn restrictor under no spiking (dotted orange curve, the same as the solid orange curve in Figure 3b), the Newborn restrictor under 15%-H spiking (solid red curve) is shifted down and passes through the global maximum. Consequently, the global maximal community function is achieved by Cycle 150 (the green circle coincides with the black star). Other legends are the same as Figure 3.

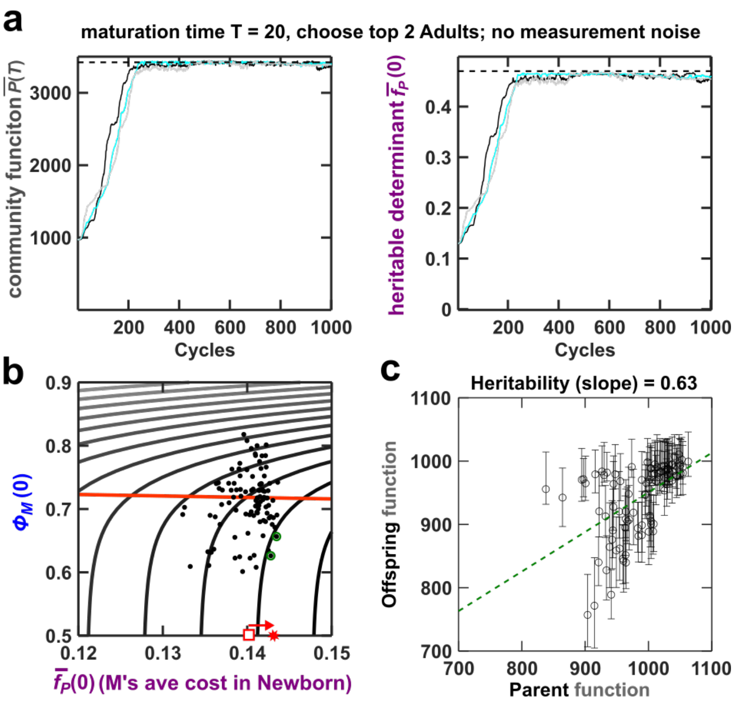

Supplementary Figure 9: Selection efficacy improved with improved heritability under maturation time $T = 20$ (longer than $T = 17$ used in most other simulations). **(a)** Evolutionary dynamics of artificial community selection. Except for the maturation time, other simulation parameters are the same as those for Figure 6a. Figure legends are the same as Supplementary Figure 2. **(b)** Visualization of inter-community selection on the landscape when maturation time $T = 20$. Figure legends are the same as Figure 5B(iii). **(c)** Heritability of community function estimated from the slope of linear regression between parent and offspring community functions. The 100 parents are the 100 communities from Cycle 24 of the simulation displayed by the black curve in (a). Circles represent the mean, and error bars extend from 25% to 75% quantile. Each circle and error bar is calculated from ~60 offspring communities. The landscape is similar to Figure 5b iii corresponding to a higher heritability, as demonstrated in (c).

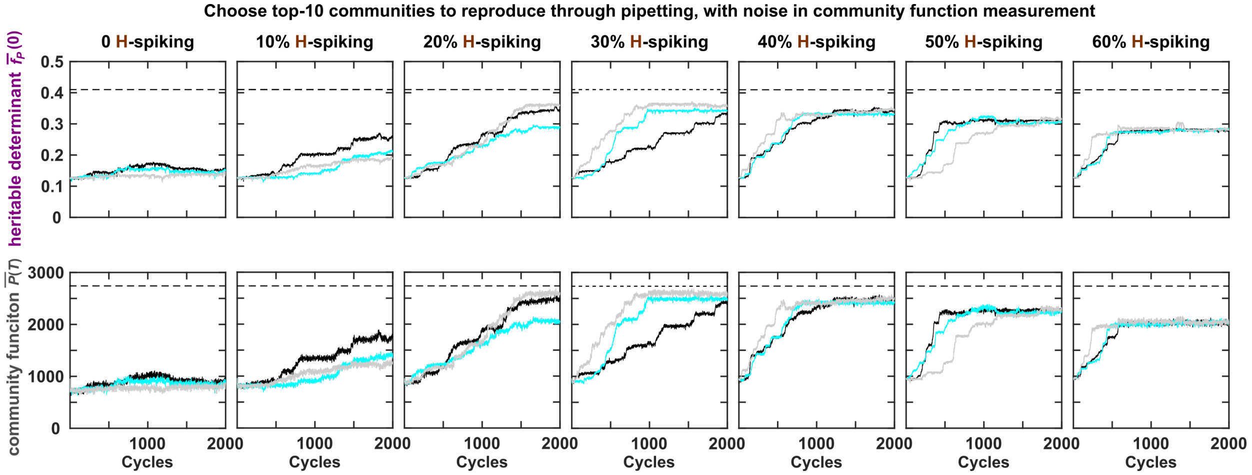

Supplementary Figure 10: Spiking H at a wide range of percentages improves selection outcome when chosen Adults are reproduced through pipetting. Adults ranking top 10 in measured community functions (= true community function + a normal random variable with zero mean and standard deviation of ~10% of the community function of Cycle 1) were chosen to reproduce. Each of the 10 chosen Adults reproduced 10 Newborns. The total biomass of a Newborn fluctuated around the target value ($BM_{\text{target}} = 100$), of which the indicated percentage was “pipetted” from H monoculture while the rest was “pipetted” from the parent Adult. Consequently, Newborn total biomass and species composition fluctuated stochastically according to sampling error. Top panels show the dynamics of heritable determinant $\overline{f}_P(0)$, the cost paid by M averaged first within and then among chosen Adults. Bottom panels show $\overline{P}(T)$, the average community function of chosen Adults. Figure legends are the same as Supplementary Figure 2.

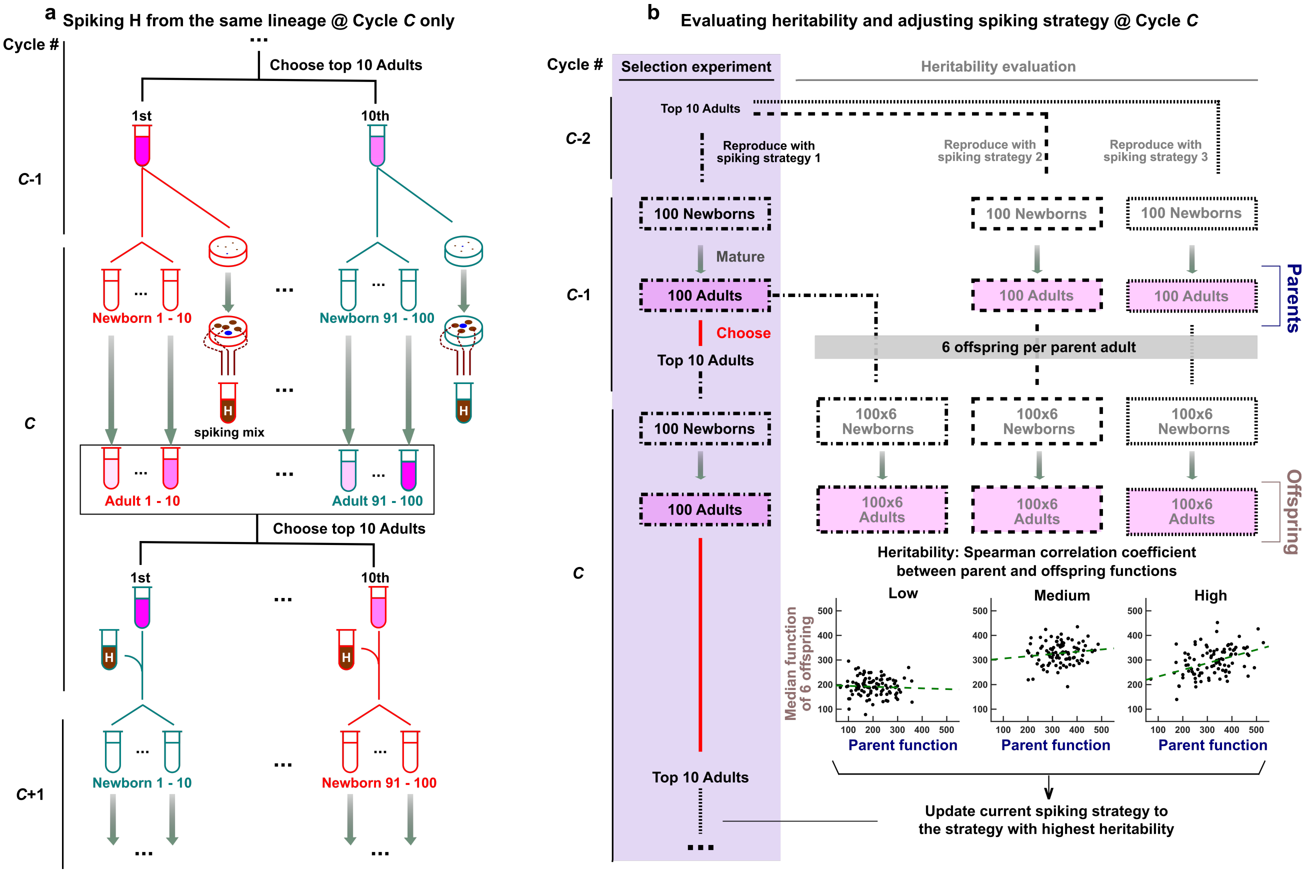

Supplementary Figure 11: Schematics of spiking based on periodic heritability checks. **(a)** Spiking during community reproduction at Cycle C with H spiking mix consisting of colonies isolated at Cycle $C-1$. Cycle $C-1$: Each chosen Adult in Cycle $C-1$ is outlined in different colors to indicate separate lineages. After each chosen Adult gives rise to 10 Newborns, the remaining Adult is plated (in simulations, this means that several H and M cells are randomly chosen from the remaining Adult). Communities and the plates share identical color outline to indicate their shared lineage. Cycle C: The 100 Newborns mature into Adults and H colonies develop on the plates. Several H colonies are randomly chosen and mixed at equal biomass as the "spiking mix". At the end of Cycle C, all Adults are ranked according to their function. To reproduce a chosen Adult, offspring Newborns are spiked with the spiking mix from the same lineage (same outline color). **(b)** Evaluating heritability and adjusting spiking strategy at Cycle C. Here we illustrate 3 candidate spiking percentages, with the left purple box highlighting the current spiking strategy. Cycle $C-2$: The 10 chosen Adults are reproduced with all 3 spiking strategies, generating 3 sets of 100 Newborns (different line styles). Cycle $C-1$: The 3 sets of 100 Newborns mature into Adults, and their functions define the "Parent function". Each Adult then gives birth to 6 offspring Newborns under the respective spiking strategy, and when these mature, the median of 6 functions defines the "Offspring function". Cycle C: At the end of cycle C, we can scatter plot the function of each parent against the median function of its 6 offspring (the median is used because it is less sensitive to outliers, which might work better for small number of offspring per parent). The heritability is estimated from the slope of the least squares regression line. In parallel, the current spiking strategy continues until Cycle C when spiking strategy is updated. In this example, the updated current strategy is spiking strategy 3.

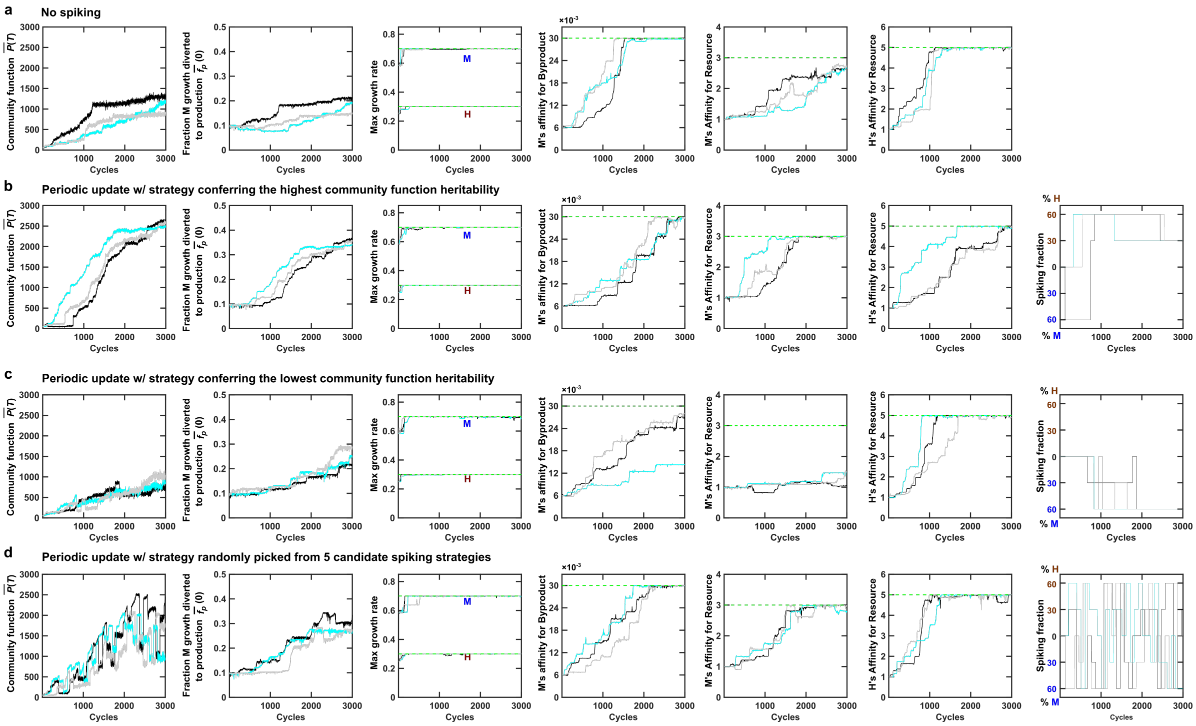

Supplementary Figure 12: Spiking based on periodic community function heritability checks boosts selection efficacy. Evolutionary dynamics of artificial community selection under no-spiking strategy **(a)** and different spiking strategies **(b-d)**. Spiking strategies conferring the highest community function heritability result in the best outcome (Compare panel b vs. panels a, c and d). The first two columns of a and b are the same as Figure 7. Spiking mix used in (b-d) are expanded from 5 clones of M or H. Black, cyan and gray curves represent 3 independent simulation replicas. The first column displays the dynamics of the community functions averaged among the 10 chosen Adults. Columns 2-6 display the dynamics of the following community function determinants averaged among the chosen communities: Newborn average cost paid by M $\overline{f}_P(0)$, Newborn average maximal growth rates of H and M $\overline{g}_{Hmax}$ and $\overline{g}_{Mmax}$, Newborn average affinity of M to Byproduct $\overline{K}_{MB}$, Newborn average affinity of M to Resource $\overline{K}_{MR}$, and Newborn average affinity of H to Resource, $\overline{K}_{HR}$. The overbar represents averaging first within each community, then across chosen communities. The last column of (b-d) displays the spiking percentage implemented in corresponding selection simulation. The green dashed lines mark the evolutionary upper-bounds of the phenotypes.

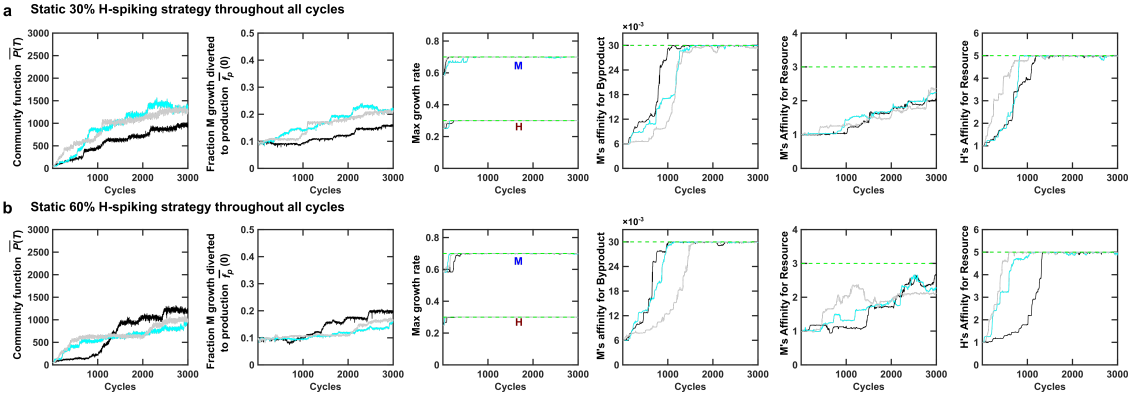

Supplementary Figure 13: Static spiking is not effective. Evolutionary dynamics of artificial community selection under **(a)** static 30%-H spiking strategy and **(b)** static 60%-H spiking. Compared to spiking based on periodic heritability check (Figure 7c), static spiking is not as effective. Figure legends are the same as Supplementary Figure 12.

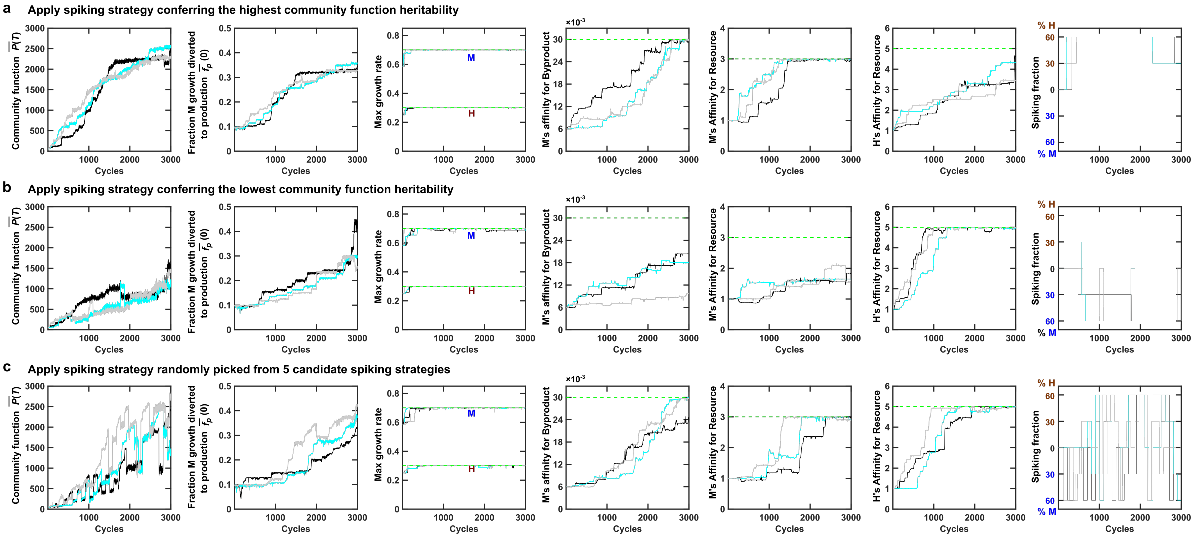

Supplementary Figure 14: Spiking with 1 clone of H or M generates qualitatively similar dynamics as spiking with 5 clones of H or M (Supplementary Figure 12). Figure legends are the same as Supplementary Figure 12.

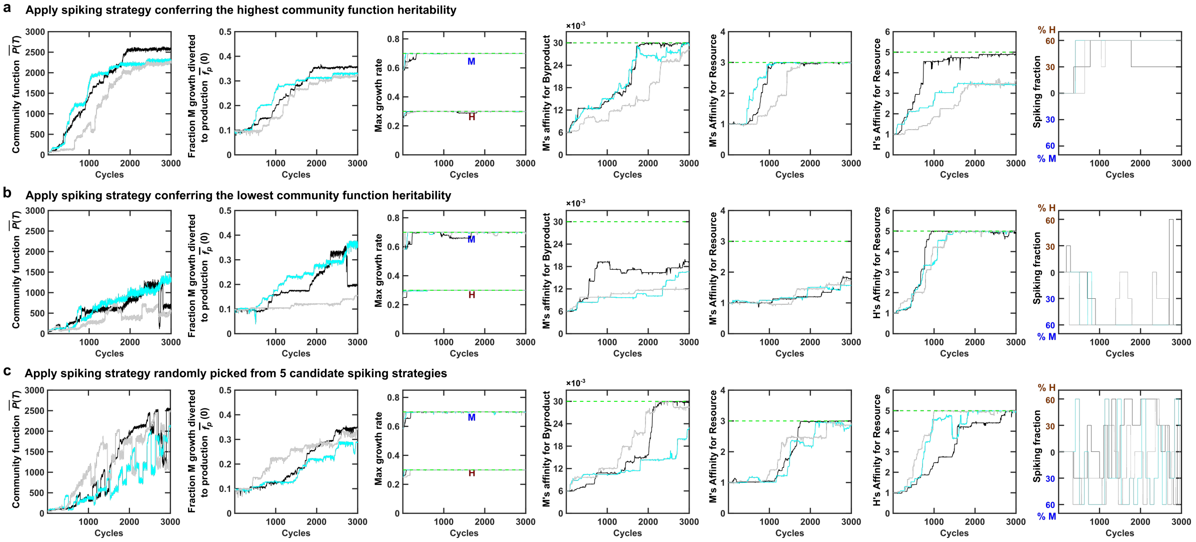

Supplementary Figure 15: Spiking with 2 clones of H or M generates qualitatively similar dynamics as spiking with 5 clones of H or M (Supplementary Figure 12). Figure legends are the same as Supplementary Figure 12.

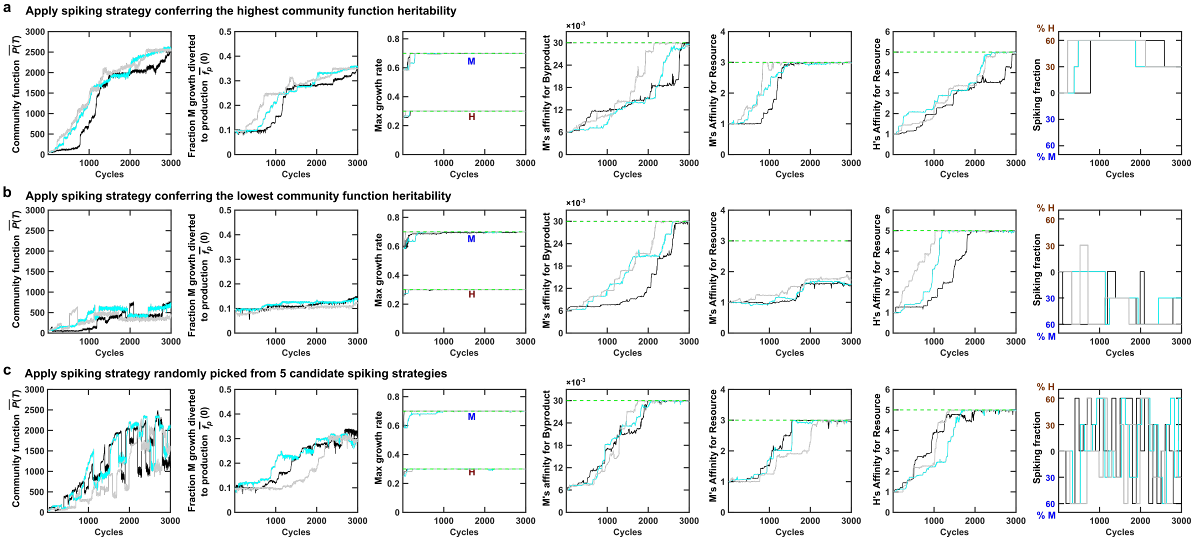

Supplementary Figure 16: Spiking with 10 clones of H or M generated qualitatively similar dynamics as spiking with 5 clones of H or M (Supplementary Figure 12). Figure legends are the same as Supplementary Figure 12.

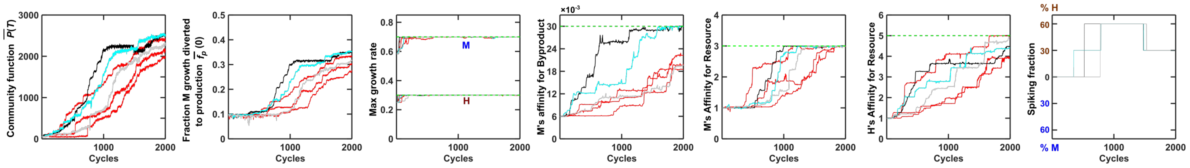

Supplementary Figure 17: Evolutionary dynamics of species spiking under "adaptive check" (black, cyan and gray) are similar to those under periodic check (red, heritability checked every 100 cycles). During adaptive check, heritability of community function under various spiking strategies is checked only when the average rate of improvement in community function over the last 50 cycles is less than zero. After every heritability check, spiking percentage conferring the highest heritability is implemented. Compared to applying spiking strategy based on periodic heritability checks, heritability is evaluated less frequently under adaptive check (~16 vs. ~8 times over 2000 cycles). The red curves are evolutionary dynamics from Figure 7c-d and Supplementary Figure 12b.

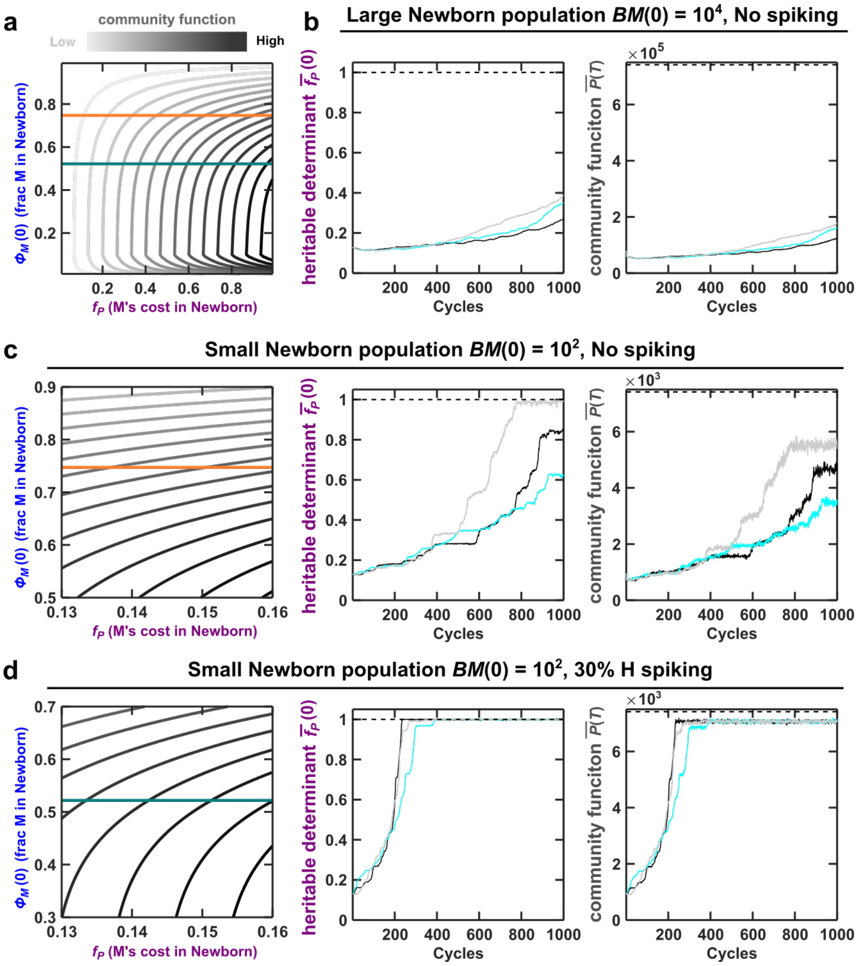

Supplementary Figure 18: Selection efficacy is deterred by large population size and facilitated by species spiking even when M does not pay a fitness cost to make the Product so that community function is not costly. Specifically, 1 replaces $(1 - f_P)$ in the first term on the right hand side of Equation 5. **(a)** Full landscape obtained by integrating Equations 1-7 with the aforementioned modification. Similar to Figure 5a ii, the orange and teal lines delineate the Newborn restrictor under no spiking and 30%-H spiking, respectively. **(b)** Evolutionary dynamics when the volume and thus the total biomass of each community is scaled up by 100 times compared to those in **(c-d)**. Since M does not pay a fitness cost to make the Product, intra-community selection does not favor M cells with low f_P. Inter-community selection favors higher community function and thus community function gradually improves. **(c)** When chosen Adults are reproduced with no spiking, the local landscape looks similar to Figure 5b ii. **(d)** When chosen Adults are reproduced with 30%-H spiking, the local landscape looks similar to Figure 5b iii, indicating a higher heritability. Indeed, community function increases faster in d compared to c. Legends for the right two columns are the same as Supplementary Figure 2.

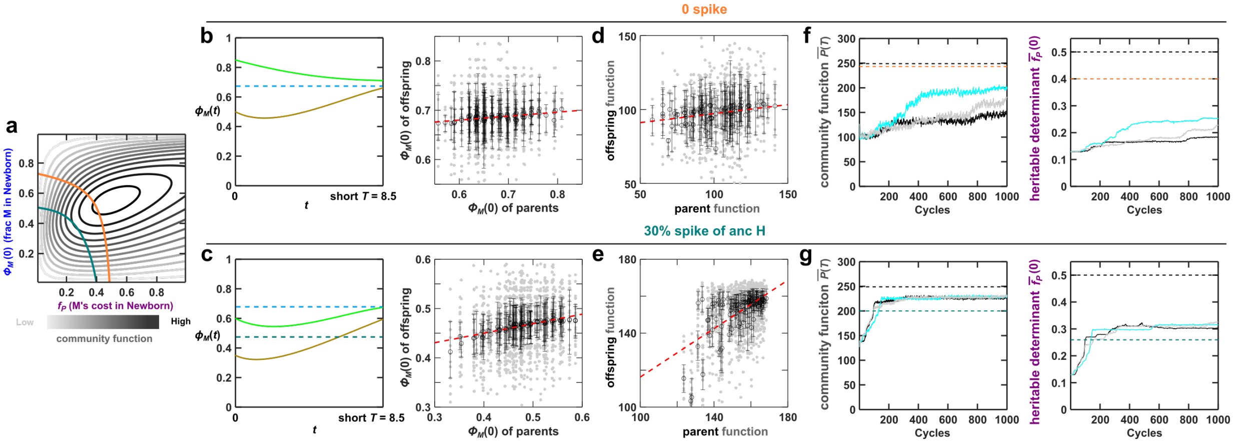

Supplementary Figure 19: Species spiking can improve selection efficacy even when maturation time is so short that species composition may not reach the steady state by the end of maturation. **(a)** Community function landscape obtained by integrating Equations 1-7 over a shorter maturation time, from $t = 0$ to $t = 8.5$ (half of that used in most other figures). Note that the landscape looks different with a shorter maturation time. The orange and teal lines delineate the Newborn restrictors under no spiking and 30%-H spiking, respectively. We consider community selection without spiking (b-f) or with 30%-H spiking (c-g). **(b, c)** Dynamics of the fraction of M biomass $\phi_M(t)$ over a short maturation cycle when $f_P = 0.18$ at different $\phi_M(0)$. The blue dashed line corresponds to the species attractor at $f_P = 0.18$. In (b), the Newborn restrictor coincides with the attractor; while in (c), the Newborn restrictor in teal dashed line is lower than the species attractor because Newborns are reproduced under 30%-H spiking. Compared to $T = 17$ (Figure 2d and Supplementary Figure 6), $\phi_M(t)$ does not always reach the blue attractor by the end of maturation. As a result, there is a slight correlation between parent communities' $\phi_M(0)$ and offspring communities' $\phi_M(0)$. In scatter plots of (b-e), circles mark the mean and error bars extend from 25% to 75% quantile. In (b, d), each circle is averaged from ~8 offspring communities. In (c, e), each circle is averaged from ~18 offspring communities. Individual data points are plotted in gray dots. **(d, e)** Heritability is increased (larger slope) with 30%-H spiking. **(f, g)** Evolutionary dynamics. The black dashed lines mark the global maximal community function and the corresponding f_P^*. The orange and teal dashed lines mark the the maximal function and the corresponding f_P along the orange and teal restrictor, respectively. Community function climbs faster along the teal restrictor (due to higher heritability) compared to along the orange restrictor, but the maximal community function achievable along the teal restrictor is lower than that along the orange restrictor. In g, community function and $\overline{f}_P(0)$ climbed higher than teal dashed lines due to fluctuations in Newborn species composition: instead of being confined onto the restrictor, the communities can access a strip along the restrictor and can thus reach a slightly higher community function. This is similar to Figure 5b (ii) and (iii) where selected communities (green circles) are slightly away from the restrictor and achieve functions higher than those expected from the restrictor.

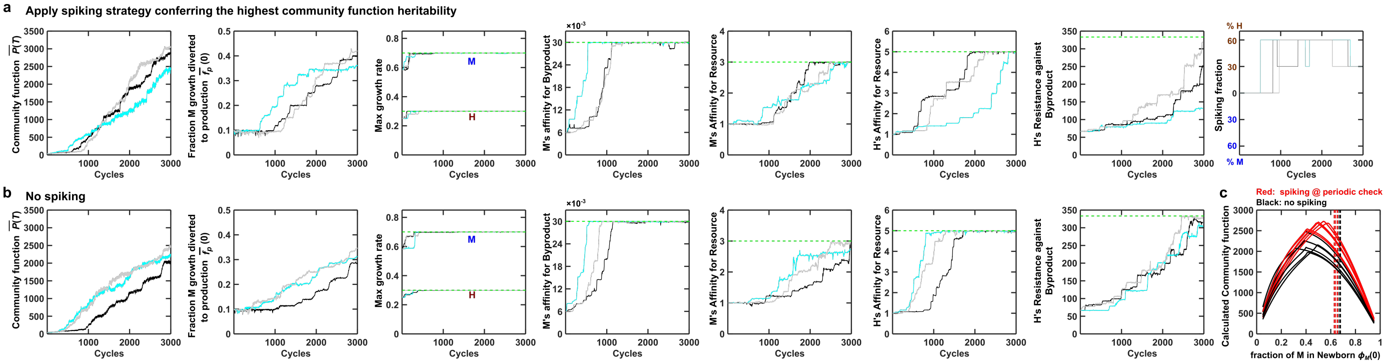

Supplementary Figure 20: Spiking based on periodic heritability checks slightly but significantly improves selection on a mutualistic community. **(a)** Evolutionary dynamics of a mutualistic community when heritability of community function under five spiking strategies (no spiking, 30%-H, 30%-M, 60%-H and 60%-M) are checked every 100 cycles, similar to simulations plotted in Figure 7(c-e) and Supplementary Figure 12b. **(b)** Evolutionary dynamics of the mutualistic community under selection with no spiking. The legends are the same as Supplementary Figure 12. **(c)** To compare selection outcomes, we focus on the highest-functioning community at the endpoint of each simulation (Cycle 3000). We calculate community functions at different Newborn species compositions (similar to Figure 7, to ensure comparable Newborn compositions), and compare community functions at the respective steady state species compositions marked by dashed lines. For this mutualistic community, community selection without spiking is already efficient. As a result, spiking based on periodic heritability check results in only a slight improvement. However, the slight improvement is significant: The 6 community functions at steady state species compositions from the red curves (spiking with periodic heritability check) are consistently higher than all 6 from the black curves (no spiking), indicating a significant difference between the two distributions (Mann-Whitney U test, $n_1 = n_2 = 6$, $p = 10^{-3}$, one-tailed). Note that the dynamics of 3 out of 6 simulations are plotted in a and b to avoid overcrowding.

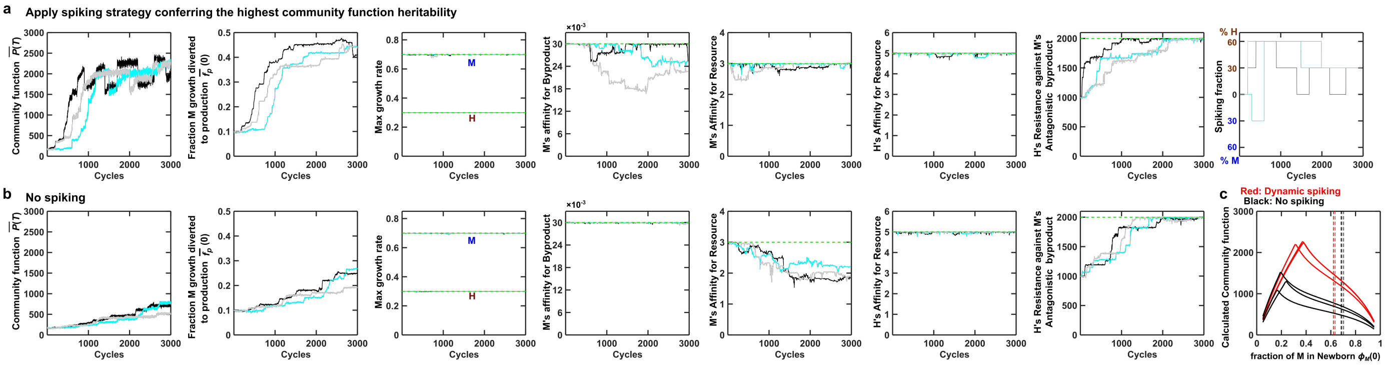

Supplementary Figure 21: Spiking based on periodic heritability checks improves selection on an exploitative community. **(a)** Evolutionary dynamics of an exploitative community when heritability of community function under five spiking strategies (no spiking, 30%-H, 30%-M, 60%-H and 60%-M) are checked every 100 cycles, similar to simulations plotted in Figure 7(c-e) and Supplementary Figure 12b. **(b)** Evolutionary dynamics of the exploitative community under selection with no spiking. The legends are the same as Supplementary Figure 12. **(c)** To compare selection outcomes, we focus on the highest-functioning communities at the endpoint of each simulation (Cycle 3000). We calculate community functions based on their heritable determinants ($\overline{f}_P(0)$, $\overline{g}_{Mmax}(0)$, $\overline{K}_{MR}(0)$, etc) using Equations 1-5, 7 and 9-10 under different $\phi_M(0)$ assuming $BM(0) = 10^2$. We also calculated the steady states ϕ_M from the same equations and plot them as dashed lines. The 3 red and 3 black curves are calculated from the 3 replica simulations in (a) and (b), respectively. Compared to communities evolved with no heritability checks or spiking (black curves), communities evolved with spiking guided by periodic heritability checks (red curves) perform better over a wide range of $\phi_M(0)$, including near the steady state species compositions (dashed lines).

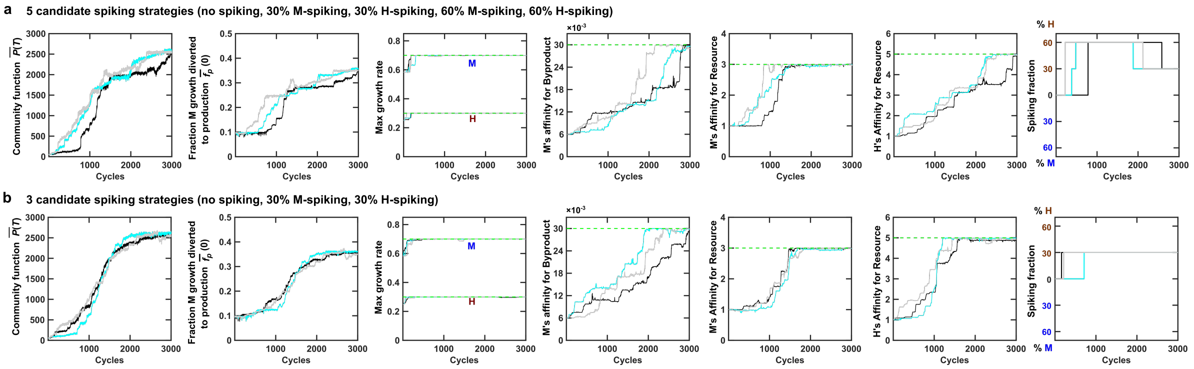

Supplementary Figure 22: Evolutionary dynamics of community selection with 5 or 3 candidate spiking strategies based on periodic heritability checks. Spiking mix consists of 10 evolved H clones or 10 evolved M clones. **(a)** is copied from Supplementary Figure 16a. **(b)** Compared to community selection with 5 candidate spiking strategies, community function increases at a slightly faster pace when there are only 3 candidate spiking strategies. Furthermore, selection with 3 candidate spiking strategies reduces the workload of heritability check by 2/5. Note that in (a), the last stretch of community function ascent (from 2000~2500) closely follows the switching of spiking strategy from 60%-H to 30%-H. The legends are the same as Supplementary Figure 12.

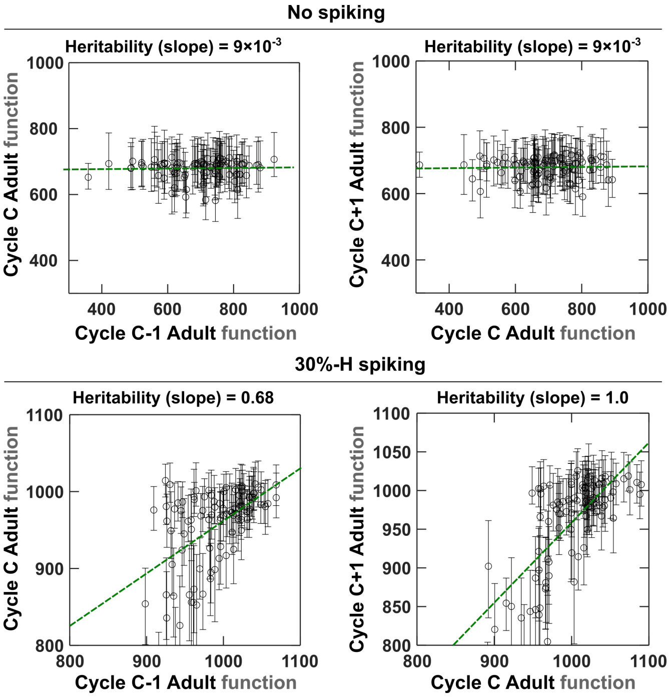

Supplementary Figure 23: Heritability of community function remains similar over two consecutive cycles. When heritability of community function is low, it does not change much over two cycles (top panels, no spiking). When heritability of community function is high, it can fluctuate over two cycles, but remains high (bottom panels, 30%-H spiking). Circles represent the mean, and error bars extend from 25% to 75% quantile. In top panels and bottom panels, each error bar is calculated from ~60 and 100 offspring communities, respectively. The 2 plots in the left column are from Figure 5c ii and iii. Communities of Cycle C are reproduced from Adults with top-2 highest functions in Cycle $C-1$. Heritability of community function is estimated from the slope of the linear regression depicted by the green dashed line.

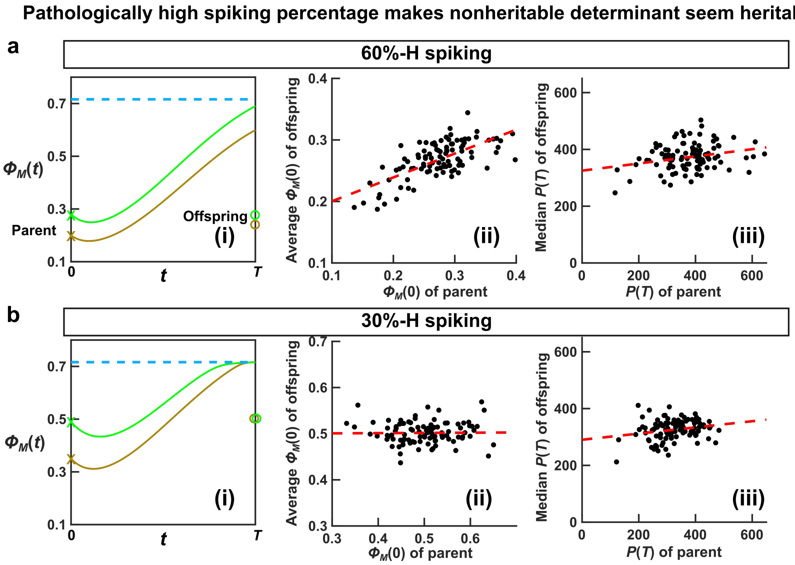

Supplementary Figure 24: Spiking a species at too high a fraction can make a nonheritable determinant partially heritable. Compare 60%-H spiking **(a)** versus 30%-H spiking **(b)**. **(i)** Species composition dynamics of two communities (green and mustard curves). Starting at Newborns immediately after spiking (crosses; parent Newborns), species compositions moves toward the attractor (blue dashed line). In 30%- but not 60%-H spiking, species composition reaches the blue attractor by the end of maturation. After reproducing with respective spiking percentages, average offspring Newborn compositions are plotted with circles. In 30%-H spiking, average offspring Newborns have the same species composition (green circle overlapping with mustard circle in b(i)). In contrast, in 60%-H spiking, average offspring Newborns have different species composition (green circle above mustard circle in a(i)). **(ii)** In 60%-H but not 30%-H spiking, the determinant $\phi_M(0)$ displays partial heritability. **(iii)** Consequently, although 60%-H spiking strategy confers similar community function heritability as 30%-H spiking strategy (similar slopes of the red dashed lines in a(iii) and b(iii)), community function improved faster under 30%-H spiking strategy (Supplementary Figure 22b) than under 60%-H spiking strategy (Supplementary Figure 22a). This is because in 60%-H spiking, a portion of community function heritability originates from the misleading heritability created by a nonheritable determinant.

References

1. Xie, L., Yuan, A. E., & Shou W. Simulations reveal challenges to artificial community selection and possible strategies for success. *PLOS Biology* **17**, e3000295, (2019).
